# Tuning of Exchange Coupling and Switchable Magnetization Dynamics by Displacing the Bridging Ligands Observed in Two Dimeric Manganese(III) Compounds

**DOI:** 10.1038/srep44982

**Published:** 2017-03-21

**Authors:** Xiang-Yu Liu, Pei-Pei Cen, Li-Zhou Wu, Fei-Fei Li, Wei-Ming Song, Gang Xie, San-Ping Chen

**Affiliations:** 1 School of Chemistry and Chemical Engineering, State Key Laboratory Cultivation Base of Natural Gas Conversion, Ningxia University, Yinchuan 750021, China; 2 Key Laboratory of Synthetic and Natural Functional Molecule Chemistry of Ministry of Education, College of Chemistry and Materials Science, Northwest University, Xi’an 710069, China; 3 College of Science, Northeast Agricultural University, Harbin 150030, China

## Abstract

Two Mn(III)-based dimers, [Mn_2_(bpad)_2_(CH_3_O)_4_]_n_ (1) and [Mn_2_(bpad)_2_(pa)_2_]_n_·2H_2_O (2) (Hbpad = N^3^-benzoylpyridine-2-carboxamidrazone, H_2_pa = phthalic acid), have been assembled from a tridentate Schiff-base chelator and various anionic coligands. Noteworthily, compound 1 could be identified as a reaction precursor to transform to 2 in the presence of phthalic acid, resulting in a rarely structural conversion process in which the bridges between intradimer Mn(III) ions alter from methanol oxygen atom with *μ*_2_-O mode in 1 (Mn 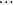 Mn distance of 3.046 Å) to *syn*-*anti* carboxylate in 2 (Mn 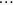 Mn distance of 4.043 Å), while the Mn(III) centers retain hexa-coordinated geometries with independently distorted octahedrons in two compounds. The dc magnetic determinations reveal that ferromagnetic coupling between two metal centers with *J* = 1.31 cm^−1^ exists in 1, whereas 2 displays weak antiferromagnetic interactions with the coupling constant *J* of −0.56 cm^−1^. Frequency-dependent ac susceptibilities in the absence of dc field for 1 suggest slow relaxation of the magnetization with an energy barrier of 13.9 K, signifying that 1 features single-molecule magnet (SMM) behavior. This work presents a rational strategy to fine-tune the magnetic interactions and further magnetization dynamics of the Mn(III)-containing dinuclear units through small structural variations driven by the ingenious chemistry.

With the fascination for molecule-based magnetic materials as the background, design and synthesis of new single-molecule magnets (SMMs) have attracted extensively attention because of their potential applications as high density memory materials, molecular switches, and so on^1^,^2^,^3^,^4^,^5^,^6^. Undoubtedly, exploring rational strategies to adjust and control the structure and magnetism of SMMs is always the active research area for ascertaining magneto-structural correlation and developing magnetic materials with excellent performance. Among the reported SMMs, Mn(III)-based dimmers of Schiff-base ligands with various organic polydentate O- or N-donors are well constructed and have been widely researched due to their potential in the field of molecule-based magnet^7^,^8^,^9^,^10^. As one of the most important research topics, perceiving the magnetic properties of dinuclear Mn(III) compounds depends on resolving the exchange interactions between the constituent metal centers composing their cores^11^,^12^,^13^. The magnetic results of the known dimeric Mn(III) compounds illustrates that some of them present intermetallic antiferromagnetic exchange whereas other compounds experience ferromagnetic intradimer interaction triggered by the combined effect of an high-spin ground state (*S*_T_ = 4) and uniaxial magnetic anisotropy *D* specified by the zero-field splitting (ZFS) constants to display the slow relaxation of the magnetization, the most performance characteristic of SMMs^14^,^15^. As reported previously, the slight structural changes may fundamentally convert the natures of magnetic interactions, further impacting on the dynamic behaviors of magnetization^16^,^17^,^18^, therefore, modulation of the intracluster magnetic interaction by altering the corresponding couplers between two Mn(III) ions is regarded as a sensible operation when the motifs of the dimeric Mn(III) units are taken into consideration.

Actually, regulating the structure and properties of compounds at molecular level still remains the particular challenge that is resulted from the reaction conditions such as pH, solvent, and temperature exerting significant effects on the structures of target compounds^19^,^20^. A handful of reports concern that the solvent molecules which feature definitely coordination ability and are facilely substituted by other organic spacers usually rouse the interesting processes of structural conversion to influence on the coordination actions of metal ions and even reverse the magnetic interactions, finally complying to the requirement for fine-tuning the structure and magnetism^21^,^22^.

Inspired by the aforesaid considerations, employing an versatility tridentate Schiff-base derivative (N^3^-benzoylpyridine-2-carboxamidrazone, Hbpad), derived from the condensation of benzoyl hydrazine and 2-cyanopyridine (Fig. 1), two new cases of dimeric Mn(III) units, [Mn_2_(bpad)_2_(CH_3_O)_4_]_n_ (**1**) and [Mn_2_(bpad)_2_(pa)_2_]_n_·2H_2_O (**2**), were synthesized with deprotonated methanol molecule and phthalic acid (H_2_pa) as bridged coligands, respectively. The crystal structures and magnetic properties of two title compounds were systematically characterized. Most interestingly, **1** undergoes a self-assembly process depending on subtle reaction condition to yield compound **2** with the methanol species interchanged by pa^2−^ anion (Fig. 2). As we expected, different architectures are responsible for the diverse magnetic behaviors. Compound **1** possesses intramolecular ferromagnetic coupling and slow magnetic relaxation of typical SMMs, while the two Mn(III) centers are antiferromagnetically exchanged in compound **2**.

## Results and Discussion

### Crystal Structure of 1

The structure of compound **1** is characterized by single-crystal X-ray diffraction analysis, it crystallizes in the monoclinic system space group *P*2_1_/c. The asymmetric unit contains one crystallographic independent Mn(III) center, one bpad^-^ anion and two CH_3_O^−^ anion. As shown in Fig. 3a, hexa-coordinated Mn(III) ion presents distorted octahedral geometry in which the equatorial plane is consist of a pyridine nitrogen atom (N1), an amidrazone nitrogen atom (N3) and a carbamoyl oxygen atom (O1) from single bpad ligand, as well as a hydroxylate oxygen atom (O2A) from the CH_3_O^−^ ligand (Mn1-N1 = 2.304(3) Å, Mn1-N3 = 2.039(3) Å, Mn1-O1 = 2.144(2) Å, Mn1-O2A = 1.941(2) Å). Out of the plane, the apical positions are occupied by two deprotonated oxygen atom (Mn1-O2 = 1.951(2) Å, Mn1-O3 = 1.855(2) Å) from two independent CH_3_O^−^ anions. Obviously, the Mn(III) ion is in distorted octahedral geometry but do not display Jahn-Teller elongation. And then, the two alcoholic oxygen atoms (O2 and O2A) with *μ*_2_-O mode act as bonders to connect neighboring Mn(III) centers, forming a Mn_2_O_2_ dimetallic pattern. The Mn-O separations in the Mn_2_O_2_ core are respectively of Mn1-O2A = Mn1A-O2 = 1.951(2) Å, Mn1-O2 = Mn1A-O2A = 1.941(2) Å, and the distance of intramolecular Mn(III) ions is 3.046 Å. Inspired by the symmetric of the Mn_2_O_2_ core, coincidentally, it appears that the *μ*_2_-O mode of the CH_3_O^−^ ligands forms a standard parallelogram. Furthermore, adjacent Mn(III) dimers are integrated to each other *via* intermolecular hydrogen-bonding composed of O3 atom with N2 atom between symmetric dinuclear Mn(III) units and *π* 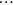 *π* stacking interaction between intercluster benzene ring and pyridine ring with the distance of 3.929 Å falling into the normal range (Fig. 3b)^23^, extending the 3D supramolecular architecture of **1**. The shortest intermolecular Mn 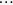 Mn distance is of 7.271 Å (Fig. 3c), predicting that the magnetic interactions between dimers may be considered to be extremely weak^7^.

### Crystal Structure of 2

**Figure 2:**
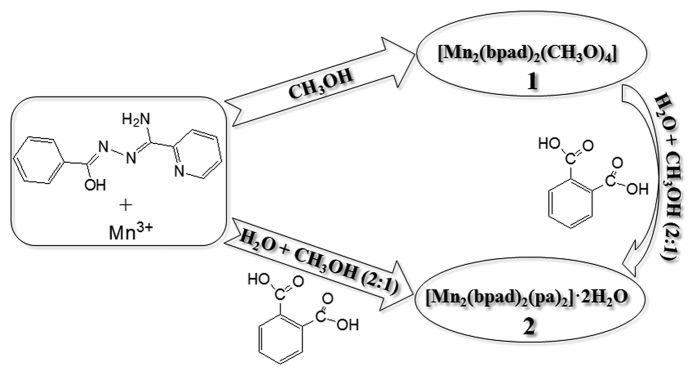
Synthesis of compounds 1 and 2.

**Figure 3:**
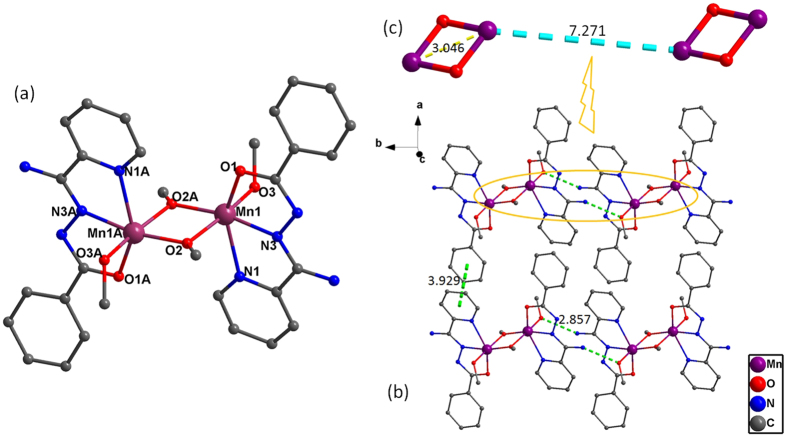
(**a**) Molecular structure of compound **1**. (**b**) Hydrogen bonding and *π*···*π* stacking in **1**. (**c**) The shortest intra- and intermolecular Mn 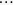 Mn distances in **1**. Hydrogen atoms are omitted for clarity.

**Figure 4:**
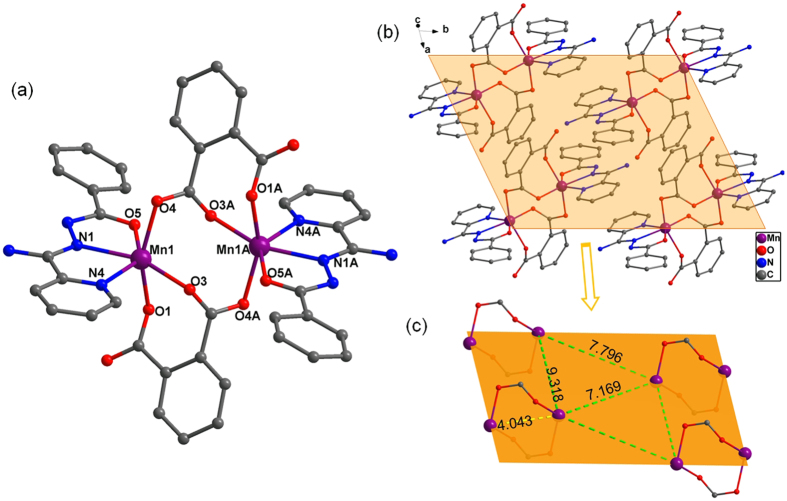
(**a**) Molecular structure of compound **2**. (**b**) Crystal packing of **2**. (**c**) The intra- and intermolecular Mn 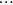 Mn distances in **1**. Hydrogen atoms and solvent water molecules are omitted for clarity.

Compound **2** crystallizes in the triclinic system with space group *P*-1 and the asymmetric unit involves one Mn(III) ion, one bpad^-^ anion, one coordinated pa^2−^ anion and one lattice water molecule. As seen in Fig. 2a, it is also a symmetric dinuclear Mn(III) ionic unit. Complexation of Mn(III) ion with bpad^-^ and pa^2−^ anions leads to a bimetallic compound with hexa-coordinated Mn(III) center as depicted in Fig. 4a. The distorted octahedral geometry of crystallographic Mn(III) ion is hold with the N_2_O_4_ group, in which the equatorial plane is bonded by a pyridyl nitrogen atom (N4), an amidrazone nitrogen atom (N1) and a carbamoyl oxygen atom (O5) from bpad ligand, as well as a carboxylate oxygen atom (O3) from pa^2−^ ligand (Mn1-O3 = 2.209(4) Å, Mn1-N4 = 2.336(6) Å, Mn1-N1 = 2.304(3) Å, Mn1-O5 = 2.275(5) Å), while two carboxylic oxygen atoms (O1, O4) from disparate pa^2−^ ligands fill into two apical positions (Mn1-O1 = 2.144(2) Å, Mn1-O4 = 2.150(5) Å). The Mn-O and Mn-N bond lengths are respectively in the ranges of 1.941–2.275 Å and 2.221–2.336 Å, which are in keeping with those observed in similar dimers^7^,^10^. The two Mn(III) ions are binded by dual *syn-anti* carboxylic motifs coming from two independent pa^2−^ ligands, producing a irregular octagon Mn_2_O_4_ core in which the Mn
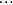
Mn distance of 4.043 Å is greater than **1**. It is observed that two carboxyl groups in one H_2_pa ligand perform monodentate coordination manner and bidentate bridging mode with *syn-anti* conformation, respectively. Also, adjacent Mn(III) dimers are combined by intermolecular hydrogen-bonding to construct the 3D supramolecular network of **2** (Fig. 4b). Likewise, the nearest distance of 7.169 Å between intercluster Mn(III) ions probably cause negligibly intermolecular magnetic interactions (Fig. 4c).

### Magnetic properties of 1 and 2

Magnetic measurements have been carried out on crystalline samples of compounds **1**–**2**, which were all pure-phase, as confirmed by PXRD (see Fig. S1 in the Supporting Information).

**Figure 5:**
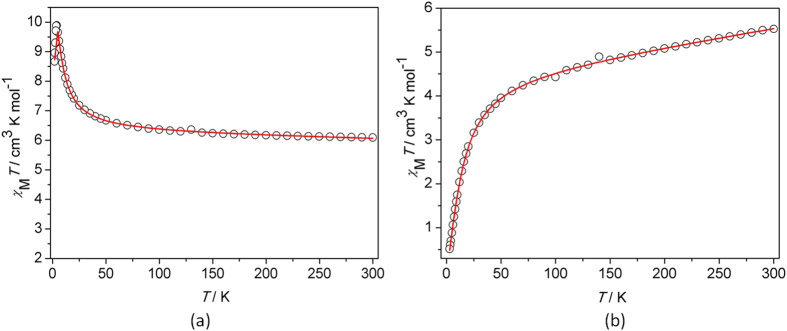
*χ*_M_*T vs T* plot for 1 and 2. The solid lines are the fit to the experimental data: (**a**) **1** (**b**) **2.**

The temperature dependence of dc magnetic susceptibility of compound **1** was collected on polycrystalline samples in the 1.9–300 K temperature range (Fig. 5a). *χ*_M_*T* value for the compound at 300 K is of 6.09 cm^3^ mol^−1^ K (per Mn_2_ unit), in good agreement with the predicted 6.0 cm^3^ mol^−1^ K for two isolated high-spin Mn(III) ions (*S* = 2). Upon lowering of the temperature, the *χ*_M_*T* value increases gradually to reach a maximum of 9.89 cm^3^ mol^−1^ K around 4 K, showing a typical ferromagnetic coupling between the Mn(III) ions. A sudden dropping of the *χ*_M_*T* value occurs for compound **1** as the temperature further decreased, which could be mainly due to the contribution of the zero-field-splitting (ZFS) from Mn(III) ion. As noticed, the *χ*_M_*T* values only shows a little of decrease in low temperature range, which illustrates no large antiferromagnetic interaction between dimmers. The susceptibility follows the Curie-Weiss law at temperatures above 15 K (Supplementary Fig. S2a), with *C* = 6.00 cm^3^ K mol^−1^ and a Weiss constant of *θ* = 5.26 K. The positive *θ* value supports the occurrence of ferromagnetic coupling between the Cu(II) centers in the compound.

For compound **2**, the product function *χ*_M_*T* keeps a slow decrease on cooling from the room temperature down to 40 K; *χ*_M_*T* (300 K) = 5.53 cm^3^ mol^−1^ K (lower than the spin-only value expected for two isolated Mn(III) ions that is 6.0 cm^3^ mol^−1^ K with *S* = 2). Then, the *χ*_M_*T* value decreases rapidly upon further cooling and ends in a value of 0.51 cm^3^ mol^−1^ K at 1.9 K, as shown in Fig. 5b. The result illuminates an exchange interaction of the antiferromagnetic nature applies between two Mn(III) ions in compound **2**. The magnetic susceptibility in the temperature range of 15 K−300 K follows the Curie−Weiss Law with a negative Weiss constant *θ* = −25.68 K and a Curie constant of 5.83 cm^3^ mol^–1^ K (Supplementary Fig. S2b). The negative *θ* value indicates the domination of the antiferromagnetic coupling between the center ions.

**Figure 6:**
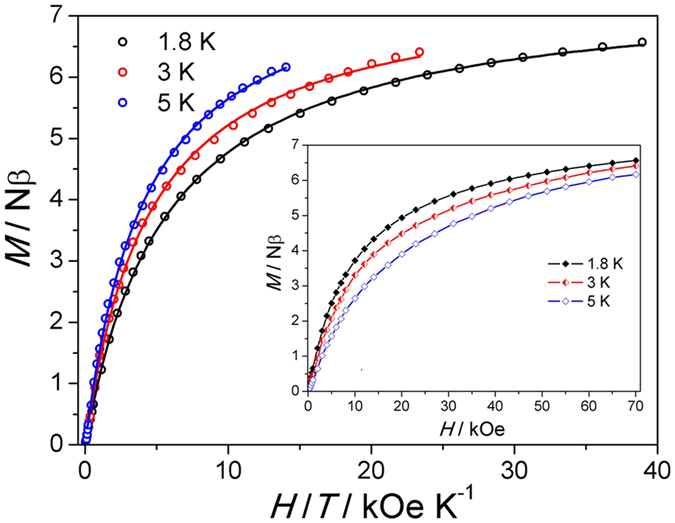
*H*/*T* dependence of Magnetization for 1. The solid lines are the best-fit curves. Inset: the Magnetization *vs. H* plot in the fields of 0–70 kOe.

The isothermal field dependent magnetizations *M(H*) at 1.8 K, 3 K and 5 K of compound **1** (Fig. 6 inset) suggests an overall ferromagnetic interaction, and the curve at 3 K of compound **2** (Supplementary Fig. S3) supports antiferromagnetic interaction in **2**, since for compound **1**, the magnetization rapidly rises with the increasing magnetic-field value to near 4.0 Nβ at 10 kOe, while the magnetization of **2** only approaches to below 1.0 Nβ at 10 kOe. The antiferromagnetic interaction observed for **2** is probably originated from the *syn-anti* bridging mode of the carboxyl group which leads to the larger intradimer Mn 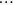 Mn distance (4.043 Å) and generally encourages antiferromagnetic coupling^24^.

The behavior is typical for dinuclear Mn(III) units that exhibit ferromagnetic or antiferromagnetic coupling between Mn(III) ions as well as the ZFS effect caused by the uniaxial anisotropy of each Mn(III) ion^12^. Therefore, the Hamiltonian given in Equation 1 can be applied to simulate the magnetic data (*S*_1_ = *S*_2_ = *S*_Mn_, *S*_*iz*_ is the *z* component of the *S*_*i*_ operator)


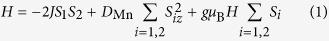


where *J* represents the magnetic coupling constant between two Mn(III) centers, *D*_Mn_ is the uniaxial anisotropy of Mn(III) ion, *g* is the *g* factor of the manganese ion. As shown in Fig. 5, the dc susceptibility was fitted by using MAGPACK package^25^. Because of the long interdimer Mn 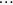 Mn separations (7.271 Å for **1** and 7.169 Å for **2**), the intermolecular magnetic interactions may be very weak according to the structural characteristics, of which the influences were neglected in fitting the data. The best fitting parameters, shown in Fig. 5 as red lines, are obtained of *g* = 1.98(1), *J* = 1.31 cm^−1^, *D*_Mn_ = −0.8 cm^−1^ for **1** and *g* = 2.02(3), *J* = −0.56 cm^−1^, *D*_Mn_ = −0.4 cm^−1^ for **2**, respectively. From the parameters, the ZFS effect, *D*_Mn_, are estimated to be the negative values of −0.8 and −0.4 cm^−1^, which is agreement with the typical range from −0.3 to −4.1 cm^−1^ for Mn(III)-based dimeric analogues reported previously^7^,^8^,^9^,^10^,^12^. It is also observed that the intramolecular coupling constant, *J*, for compound **1** is 1.31 cm^−1^, which is corresponding to the ferromagnetic exchange between Mn(III) ions and is comparable with those of ferromagnetic Mn(III) dimmers^7^,^8^,^9^, while the negative *J* value for compound **2** declares for the antiferromagnetic coupling between Mn(III) ions. As a result, the *J* value for **1** is in the same magnitude of *D* value, indicating the magnetic interaction is not neglectable in this case, which is different from the Mn_12_ system^1^.

In accordance with the magnetic behavior of known SMMs, such as [Mn_2_(saltmen)_2_(ReO_4_)_2_]^7^, [Mn(saltmen)(N_3_)]_2_^9^ and so on, the ground state (*S*_T_ = 4) is applied to fit the plot of *M* vs *H*/*T* at different temperatures by the least-squares for compound **1** (Fig. 6). The best-fit *D*_Mn_ value for **1** is −0.73 cm^−1^ (solid lines in Fig. 4), generating the theoretical energy barrier (∆/*k*_B_) of 16.8 K, where ∆/*k*_B_ = |*D*_Mn_|*S*_T_^2^/*k*_B_ (*k*_B_ expresses the Boltzmann constant). The fitting value is in agreement with that expected from the simulated variable temperature dc magnetic susceptibility.

**Figure 7:**
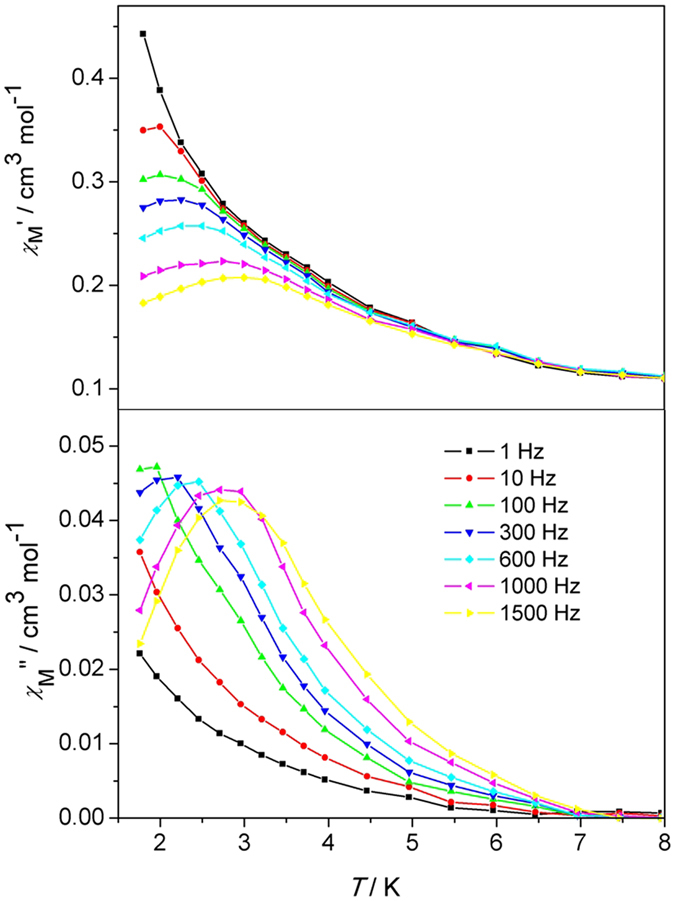
In-phase (*χ*′_M_) and out-phase (*χ*″_M_) ac magnetic susceptibility *vs T* plots for 1 under a zero dc field.

**Figure 8:**
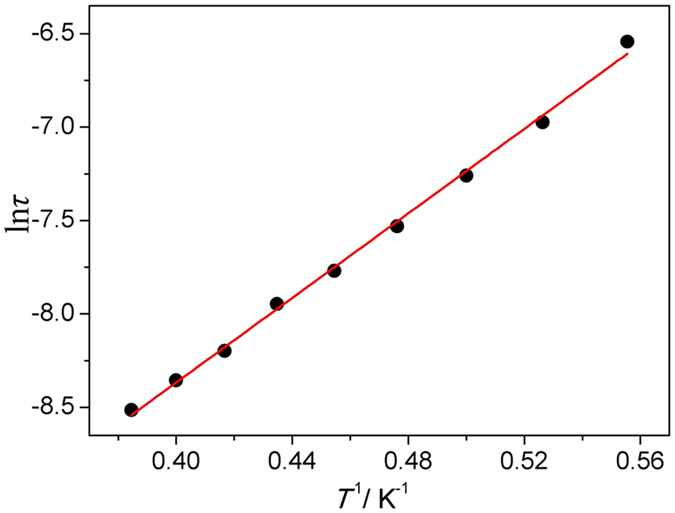
The relaxation time of the magnetization ln(*τ*) against *T*^−1^ for 1. The solid line represents the Arrhenius fit.

The measurement of the zero-field alternating current (AC) magnetic susceptibility with the oscillating field of 2.0 Oe in the range 1.8–8 K and frequencies of 1, 10, 100, 300, 600, 1000 and 1500 Hz was performed on polycrystalline samples of compound **1**. As seen in Fig. 7, a strong out-of-phase signal appears in the *χ*″_M_ vs *T* plots at 7 K, The in-phase and out-of-phase maxima peaks both could be observed above 1.8 K and show frequency dependence, as expected for SMMs, exhibiting single slow magnetic relaxation process. The peak temperatures *T*_p_ in *χ*″_M_ shifts from 1.87 to 2.92 K with frequency from 100 to 1500 Hz. The shift parameter *Ф* = (Δ*T*_P_/*T*_P_)/Δ(log *f*) = 0.3 means the slow dynamics of magnetization and corresponds with the typical range (0.1 ≤ *Ф* ≤ 0.3) of the superparamagnetic materials^26^,^27^. Furthermore, the frequency dependencies of the ac susceptibility for compound **1** were characterized under zero dc field at various temperatures (Supplementary Fig. S4), the peaks of the *χ″* ac susceptibility gradually move from low frequency to high frequency, explicating that the *χ″* of compound **1** always display frequency dependence in selected temperature range. The relaxation time *τ* value has been obtained depending on the frequency dependent ac susceptibility and derived from the *χ″* peaks following the Arrhenius equation *τ* = *τ*_0_exp(*E*_a_/*k*_B_*T*) (The relaxation time *τ* = 1/2*πf* is derived from the *χ*″_M_ vs *T* curves, *T* is the temperature at which the peak of the *χ*″_M_ appeared under different frequencies) (Fig. 8). The deduced pre-exponential factor *τ*_0_ = 2.5 × 10^−6^ s clearly confirms that **1** displays the characteristic of SMM behavior (*τ*_0_ = 10^−6^ to 10^−13^ s)^28^. An activation energy *E*_a_/*k*_B_ of 11.3 K is also worked out following the equation above, which is slightly lower than the theoretical ones calculated from *D*_Mn_ and *S*_T_ parameters (∆/*k*_B_ = |*D*_Mn_|*S*_T_^2^/*k*_B_ = 16.8 K). Note that this result may be affected by the non-dominant existence of a small amount of the quantum tunneling for magnetic relaxation (QTM) at a zero field. The QTM can lower the energy barrier, as seen in other similar type of SMM systems with isolated Mn(III)-containing dimers^7^,^8^,^9^. Further attempt to verify the conjecture above by hysteresis loop measurement within ±2 T at 2.0 K was unsuccessful, a narrow gap between the increasing and decreasing values observed in the hysteresis loop plots should be from the systematic error of SQUID because the measurement is not below blocking temperature (Supplementary Fig. S5). Field-cooled (FC) and zero-field-cooled (ZFC) magnetization experiment for **1** was also carried out, the FC and ZFC curves don’t reach a maximum and diverge above 2 K (Supplementary Fig. S6), implying the lowered blocking temperature.

In addition, the AC magnetic susceptibility experiment with a zero static field for **2** were measured at low temperature, as predicted, no out-of-phase (*χ*″_M_) signals were observed until the temperature down to 1.8 K (Supplementary Fig. S7).

## Discussion

Two dinuclear Mn(III) compounds **1** and **2** are obtained by using the handily solution evaporation method. **1** either could produce **2** with a structural alteration by the introduction of phthalic acid. Note that the reaction solvent is pure methanol for the formation of **1**, whereas mixed solvent of distilled water and methanol (2:1) for the synthesis of **2**. Structurally, for **1**, the methanol molecule as secondary ligand in which the oxygen atom with *μ*_2_-O mode bridges two Mn(III) ions (Mn 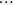 Mn = 3.046 Å) constructing the bimetallic unit. Compared with **1**, the two metal centers (Mn 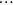 Mn = 4.043 Å) in **2** are linked by the carboxylic group with *syn*-*anti* configuration from H_2_pa coligand which crowds out the coordinated methanol anion resulting from the relatively small proportion methanol in the mixed solution. Additionally, the nearest distances between intermolecular Mn(III) ions in two compounds are roughly accordant (7.271 Å for **1**, 7.169 Å for **2**). As observed, although the Mn(III) ions are provided with six-coordinated surrounds in title compounds, the integral structures of **1** and **2** are entirely different, foreseeing discrepant magnetic behaviors. For **1**, the pathway of magnetic transmission between the dinuclear manganese unit is supplied by doubly alcoholic oxygen bridges with *μ*_2_-O manner, contributing to intradimer ferromagnetic exchange with the coupling constant (*J*) of 1.31 cm^−1^ and slow relaxation of the magnetization under zero static field, one of the important characteristics of SMMs. In contrast, the antiferromagnetic coupling with the *J* parameter of −0.56 cm^−1^ between Mn(III) ions in **2** is guided by two *syn*-*anti* carboxylic oxygen atoms. In general, it is concluded that ferromagnetic dinuclear Mn(III) unit may display SMM behavior, and the bonders between two metal ions and their coordination mode possibly are the major factors determining if the coupling interaction is ferromagnetic or antiferromagnetic. Moreover, the magnetic anisotropy of Mn(III) ion in compound **1** manifests as a negative ZFS parameter that probably plays an indispensable role in the relaxation process of a magnet and leads to excitingly dynamic magnetic behavior. Although it is still hard to define the correlation between the Mn(III) 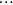 Mn(III) magnetic interaction and the structural characters of the dimeric Mn(III) compounds, it may be deduced that the intermetallic magnetic interaction would be tuned by varying the bridging ligands^29^.

## Conclusions

In present work, two new Mn(III) dimmers with a Schiff-base ligand and different bridging coligands have been successfully synthesized in different solvent conditions, as well as structurally and magnetically investigated. Noteworthily, compound **2** could be also obtained from compound **1** by replacing the bridging ligands, in respond to this, the most striking contrast in the structures of **1** and **2** is that intradimer two Mn(III) ions are bridged by *μ*_2_-O methanol oxygen atom and *syn*-*anti* carboxyl group, respectively. Structural changes give rise to drastically different magnetic properties in two title compounds. Compound **1** indicates ferromagnetic coupling between two metal ions and frequency dependence of the ac susceptibility, unambiguously behaving as a SMM. Conversely, compound **2** features intramolecular antiferromagnetic exchange and the absence of characteristic SMM behavior. Thus, the exchange coupling is provided with an important and complex influence on the overall magnetic behavior. The present results not only enrich the SMM studies, but also example the fine-tuned structures accompanying with switchable magnetic properties at the molecular level based on concise chemical process. In addition, the resulting dinuclear Mn(III) compounds would be considered as ideal building blocks to construct versatile architectures by assembling other magnetic sub-units.

## Methods

### Materials

All reagents and solvents were commercially available and used as received without further purification. The ligand, N^3^-benzoylpyridine-2-carboxamidrazone (Hbpad), was prepared from benzoyl hydrazine and 2-cyanopyridine according to the reference^30^.

### Synthesis of Hbpad ligand

A mixture of 2-cyanopyridine (3.0 g) and sodium (0.15 g) was added to the absolute methanol with stirring for 2 h, and then, benzoyl hydrazine (3.0 g) and glacial acetic acid (0.3 g) were added in the solution above. The resulting pale yellow precipitate was filtered and cleansed fully with ethanol. Drying in vacuum the analytically pure product was collected as white powder (yield 82%). Elemental analysis (%): Calcd. for C_13_H_12_N_4_O (240.29): C, 64.98; H, 5.04; N, 23.32%. Found: C, 64.92; H, 5.01; N, 23.27%. Mp: 206–207 °C; ^1^HNMR (400 MHz, DMSO-d_6_, 25 °C, ppm): *δ* = 8.58 (d, 1H, Pz-H^2^), 7.42–7.43 (dd, 1H, Pz-H^3^), 8.83–8.86 (dd, 1H, Pz-H^4^), 7.59 (d, 1H, Pz-H^5^), 7.90–7.95 (m, 2H, Ph-H^2^/H^6^), 7.43–7.48 (m, 2H, Ph-H^3^/H^5^), 7.52–7.54 (dd, 1H, Ph-H^4^), 2.44 (br s, 2H, NH_2_). Selected IR data (KBr, cm−1): 3405 (s), 3345 (m), 3214 (s), 1669 (s), 1636 (s), 1613 (s), 1589 (m), 1555 (s), 1479 (s), 1397 (s), 1298 (m), 1154 (w), 1053 (w), 997 (m), 792 (m), 697 (m).

### Synthesis of [Mn_2_(bpad)_2_(CH_3_O)_4_]_n_ (1)

A methanol solution (30 mL) of Mn(OAC)_3_∙2H_2_O (0.0536 g, 0.2 mmol) was mixed with triethylamine (0.28 ml, 2 mmol). Then, the Hbpad ligand (0.0274 g, 0.2 mmol) was added into the solution above with continuously stirring for 3 h at normal temperature. After filtration, the filtrate was slowly evaporated at room temperature. Yellow crystals of **1** were obtained on the bottom of beaker within three weeks. Yield: 46% (based on Mn^3+^). Elemental analysis (%): Calcd. for C_30_H_34_Mn_2_N_8_O_6_ (%): C, 50.52; H, 4.77; N, 15.72. Found: C, 50.48; H, 4.75; N, 15.69. IR (cm^−1^, KBr): 3387 (w), 3109 (w), 2814 (w), 2795 (m), 1638 (s), 1585 (s), 1565 (m), 1515 (s), 1476 (s), 1404 (s), 1370 (s), 1291 (w), 1170 (w), 1053 (m), 790 (w), 697 (m), 667 (m), 545 (m).

### Synthesis of [Mn_2_(bpad)_2_(pa)_2_]_n_·2H_2_O (2)

The compound **2** was prepared from two ways as follows: (i) The similar procedure described above for **1**, but simultaneously added Hbpad (0.0274 g, 0.2 mmol) with phthalic acid (H_2_pa) (0.0334 g, 0.2 mmol) as mixed-ligand into aqueous methanol (30 mL, 2:1) and stirred the solution in a semi-closed beaker for 3 hours. Pale yellow crystals of **2** were collected and the yield is ca. 58.2% based on Mn^3+^. Elemental analysis (%): Calcd. for C_42_H_34_Mn_2_N_8_O_12_ (%): C, 52.91; H, 3.57; N, 11.76. Found: C, 52.88; H, 3.55; N, 11.73. IR (cm^−1^, KBr): 3382 (m), 3104 (w), 2319 (w), 1656 (s), 1639 (s), 1574 (s), 1565 (m), 15085 (s), 1468 (s), 1434 (s), 1386 (m), 1352 (s), 1287 (m), 1164 (w), 1050 (w), 788 (m), 739 (m), 706 (m), 420(m). (ii) Compound **1** was added in **t**he mixed solution (aqua: methanol = 2:1) of phthalic acid (H_2_pa) (0.0334 g, 0.2 mmol), and stirred in a semi-closed beaker for 3 hours. After filtration, slow evaporation at room temperature of the filtrate also generated the crystals of compound **2** (Fig. 2).

### Crystallographic data collection and refinement

The single-crystal X-ray experiment was carried out on a Bruker SMART APEX-CCD-based diffractometer using graphite-mono-chromated Mo Kα radiation (*λ* = 0.71073 Å) using *ω* and *φ* scan mode. The data integration and reduction were processed with SAINT software. An empirical absorption corrections were implemented using SADABS program^31^. Data processing was accomplished with the SAINT processing program. The structure was determined by the direct methods using the SHELXS program of the SHELXTL-97^32^ package and refined with SHELXL-97^33^. The positions of the hydrogen atoms were refined isotropically for whole framework. All non-hydrogen atoms were refined anisotropic displacement parameters. Additionally, other details of crystal data, data collection parameters and refinement statistics for **1** and **2** are summarized in Table S1. Selected bond lengths and bond angles, and the hydrogen bonds of two compounds are listed in Tables S2–S5, respectively.

### Physical measurements

^1^H nuclear magnetic resonance (NMR; Beijing Oxford Instrument Company, China) spectra were carried out with a Varian 400 spectrometer utilizing tetramethylsilane as an internal standard. NMR spectra were achieved from solutions in dimethyl sulfoxide (DMSO). Elemental analysis (C, H, N) was accomplished on a Perkin-Elmer 2400 CHN elemental analyzer. The FT-IR spectra were recorded in the range of 400–4000 cm^−1^ by using KBr pellets on an EQUINOX55 FT/IR spectrophotometer. The phase purity of the polycrystalline or bulk samples were confirmed by powder X-ray diffraction (PXRD) experiments performed on a Rigaku RU200 diffractometer at 60 kV, 300 mA and CuKα radiation (*λ* = 1.5406 Å), with a scan speed of 5° min^−1^ and a step size of 0.02° in 2*θ*. Magnetic measurements were implemented on polycrystalline samples (52.57 mg for **1,** 22.19 mg for **2**) using a Quantum Design MPMS-XL7 SQUID magnetometer (restrained in eicosane to prevent torqueing at high fields). The measured susceptibilities were corrected for the diamagnetism of the constituent atoms (Pascal’s tables).

## Additional Information

**Accession codes**: X-ray crystallographic data for compounds 1 and 2 in CIF format can be obtained free of charge from the Cambridge Crystallographic Data Centre via www.ccdc.cam.ac.uk/data_ request/cif. CCDC numbers for 1 and 2, 1478578 and 1478581.

**How to cite this article:** Liu, X.-Y. *et al*. Tuning of Exchange Coupling and Switchable Magnetization Dynamics by Displacing the Bridging Ligands Observed in Two Dimeric Manganese(III) Compounds. *Sci. Rep.* **7**, 44982; doi: 10.1038/srep44982 (2017).

**Publisher's note:** Springer Nature remains neutral with regard to jurisdictional claims in published maps and institutional affiliations.

## Supplementary Material

Supplementary Information

## Figures and Tables

**Figure 1:**
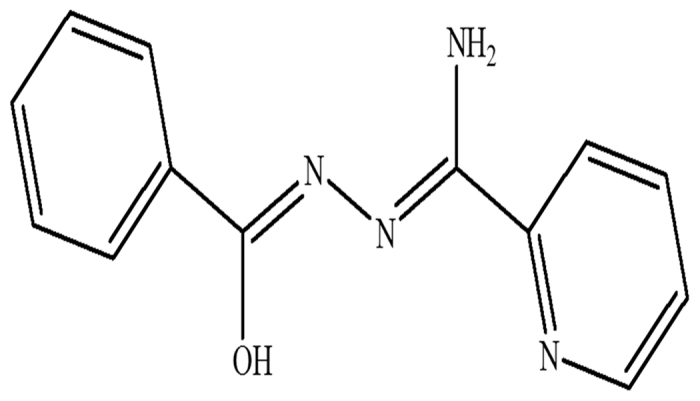
N^3^-benzoylpyridine-2-carboxamidrazone (Hbpad).
