# Supplementary material for: Tuning of Exchange Coupling and Switchable Magnetization Dynamics by Displacing the Bridging Ligands Observed in Two Dimeric Manganese(III) Compounds
Source: Sci Rep. 2017 Mar 21;7:44982. doi: 10.1038/srep44982 (PMC5359551; doi:10.1038/srep44982)
Supplement: Supplementary Information [file srep44982-s1.pdf]

Supporting Information

Tuning of Exchange Coupling and Switchable Magnetization Dynamics by Displacing the Bridging Ligands Observed in Two Dimeric Manganese(III) Compounds

Xiang-Yu Liu*,[1], Pei-Pei Cen[1,2], Li-Zhou Wu[3], Fei-Fei Li[1], Wei-Ming Song[1], Gang Xie[2] & San-Ping Chen*,[2]

[1] School of Chemistry and Chemical Engineering, State Key Laboratory Cultivation Base of Natural Gas Conversion, Ningxia University, Yinchuan 750021, China

[2] Key Laboratory of Synthetic and Natural Functional Molecule Chemistry of Ministry of Education, College of Chemistry and Materials Science, Northwest University, Xi'an 710069, China

[3] College of Science, Northeast Agricultural University, Harbin 150030, China

Contents

Figure S1. PXRD patterns for compounds: (a) **1**, (b) **2**.

Figure S2. $1/\chi_M$ *vs T* plots for **1** (a) and **2** (b), the red solid line is the best fit to the Curie-Weiss law.

Figure S3. Magnetization *vs*. H plot for **2**.

Figure S4. Out-of-phase (χ'') signal *vs*. frequency (v) plots for **1.**

Figure S5. Hysteresis loop for **1** at 2.0 K.

Figure S6. ZFC and FCM curves for **1**.

Figure S7. In-phase (χ'_M) and out-phase (χ''_M) ac magnetic susceptibility *vs T* plots for **2** under a zero dc field.

Table S1 Crystal data and structure refinement summary for compounds **1** and **2**.

Table S2 Selected bond lengths (Å) and bond angles (°) for **1**.

Table S3 Hydrogen-bonding interactions for **1**.

Table S4 Selected bond lengths (Å) and bond angles (°) for **2**.

Table S5 Hydrogen-bonding interactions for **2**.

***Corresponding author**

Dr. Xiang-Yu Liu

E-mail: xiangyuliu432@126.com, l_xy@nxu.edu.cn

***Corresponding author**

Prof. San-Ping Chen

E-mail: sanpingchen@126.com

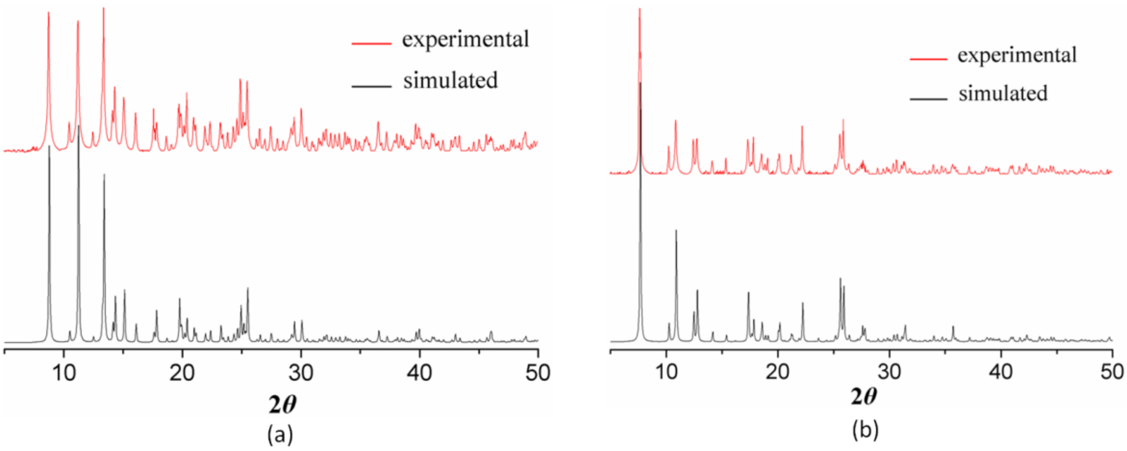

Figure S1. PXRD patterns for compounds: (a) **1**, (b) **2**.

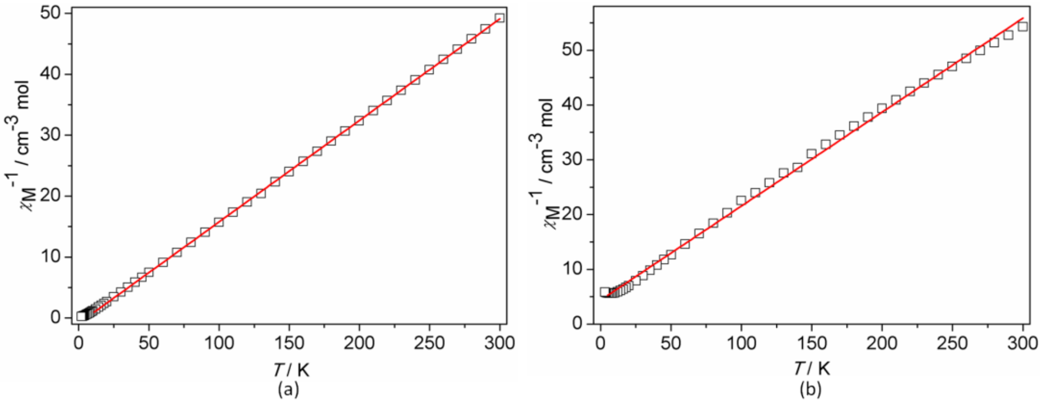

Figure S2. $1/\chi_M$ *vs* T plots for **1** (a) and **2** (b), the red solid line is the best fit to the Curie-Weiss law.

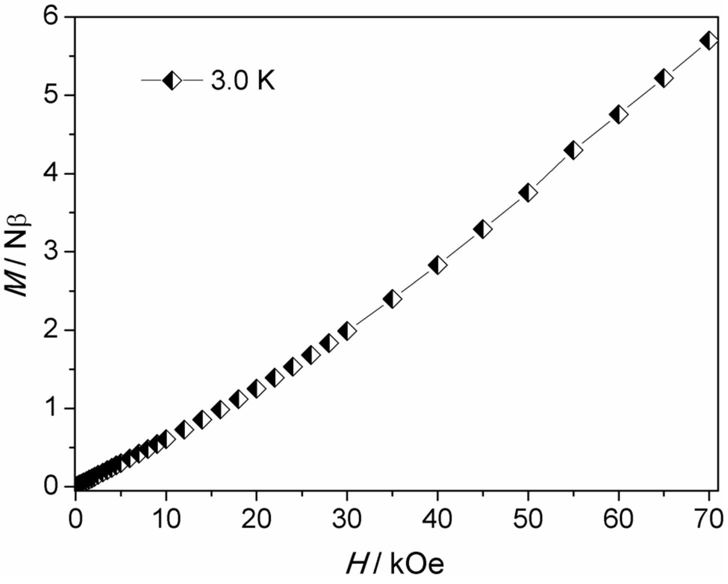

Figure S3. Magnetization *vs*. H plot for **2**.

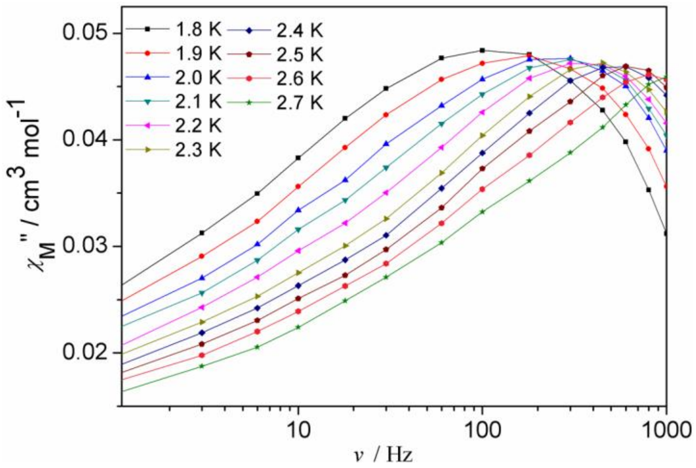

Figure S4. Out-of-phase (χ'') signal *vs*. frequency (ν) plots for **1.**

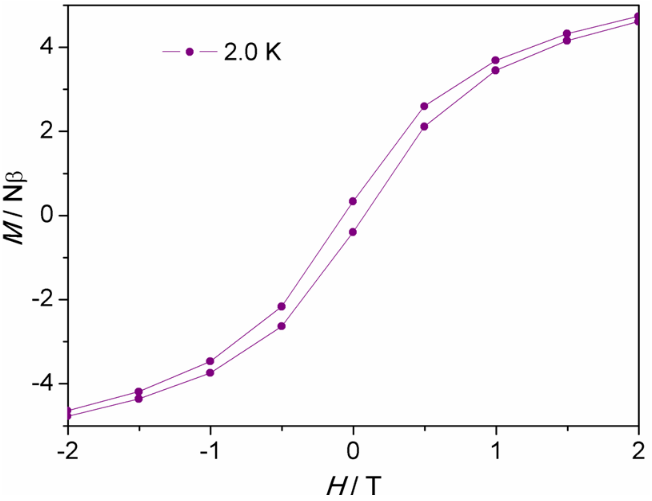

Figure S5. Hysteresis loop for **1** at 2.0 K.

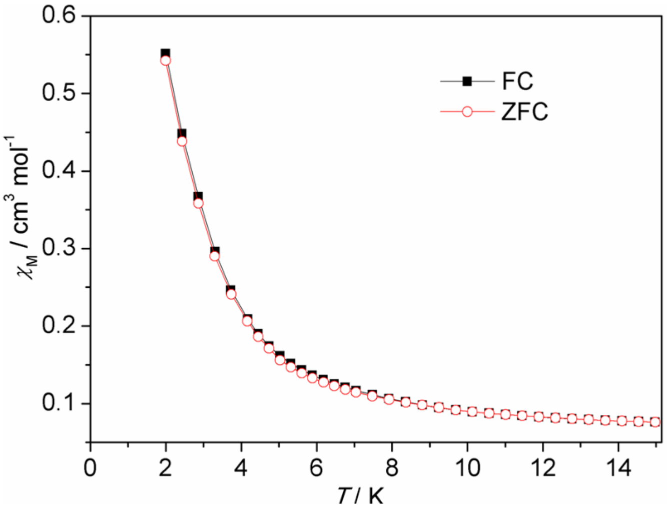

Figure S6. ZFC and FCM curves for **1**.

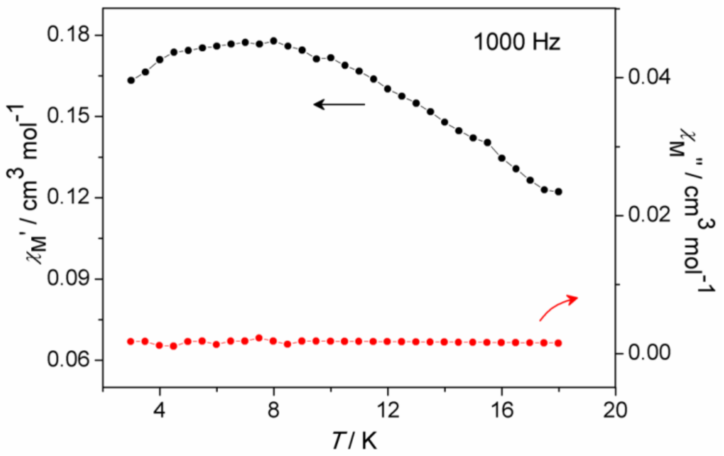

Figure S7. In-phase (χ'_M) and out-phase (χ''_M) ac magnetic susceptibility *vs* T plots for **2** under a zero dc field.

Table S1 Crystal data and structure refinement summary for compounds **1** and **2**.

	**1**	**2**
Empirical formula	$C_{30}H_{34}Mn_2N_8O_6$	$C_{42}H_{34}Mn_2N_8O_{12}$
Formula weight	712.53	952.65
Crystal system	Monoclinic	Triclinic
Space group	P21/c	P-1
a / (Å)	10.1330(9)	9.318(3)
b / (Å)	9.9394(9)	10.518(3)
c / (Å)	15.8319(13)	11.659(4)
α / (°)	90	80.484(6)
β / (°)	97.043(2)	86.012(7)
γ / (°)	90	67.526(5)
V (Å^3)	1582.5(2)	1041.3(6)
Z	2	1
Calculated density D_e / mg · m^{-3}	1.495	1.519
Absorption coeff.(μ)mm^{-1}	0.854	0.681
F(000)	736	488
R(int)	0.0357	0.0688
Θ range / (°)	2.42 to 25.00	1.77 to 25.00
Reflections collected / unique	7822/2782	5138 / 3624
Parameters refined	210	289
Final R indices [I>2σ(I)]	R_1 = 0.0406, ωR_2 = 0.1034	R_1=0.1021,ωR_2 = 0.2214
R indices (all data)	R_1 = 0.0617, ωR_2 = 0.1159	R_1=0.1915,ωR_2 = 0.2630
Temp.(K)	296(2)	296(2)

Table S2 Selected bond lengths (Å) and bond angles (°) for **1**.

**1**			
Mn(1)-O(3)	1.855(2)	Mn(1)-O(2)#1	1.941(2)
Mn(1)-O(2)	1.951(2)	Mn(1)-N(3)	2.039(3)
Mn(1)-O(1)	2.144(2)	Mn(1)-N(1)	2.304(3)
O(1)-C(7)	1.287(4)	O(2)-C(14)	1.419(3)
O(2)-Mn(1)#1	1.941(2)	O(3)-C(15)	1.423(4)
N(1)-C(1)	1.327(4)	N(1)-C(5)	1.347(4)
N(2)-C(6)	1.336(4)	N(3)-C(6)	1.310(4)
N(3)-N(4)	1.390(3)	N(4)-C(7)	1.325(4)
C(1)-C(2)	1.379(5)	C(2)-C(3)	1.369(6)
C(7)-C(8)	1.481(4)	C(8)-C(9)	1.385(4)
O(3)-Mn(1)-O(2)#1	94.63(9)	O(3)-Mn(1)-O(2)	171.13(9)
O(2)#1-Mn(1)-O(2)	77.01(9)	O(3)-Mn(1)-N(3)	92.68(10)
O(2)#1-Mn(1)-N(3)	169.02(10)	O(2)-Mn(1)-N(3)	95.18(9)
O(3)-Mn(1)-O(1)	94.67(9)	O(2)#1-Mn(1)-O(1)	113.09(8)
O(2)-Mn(1)-O(1)	91.41(8)	N(3)-Mn(1)-O(1)	74.38(9)
O(3)-Mn(1)-N(1)	90.22(9)	O(2)#1-Mn(1)-N(1)	98.81(9)
O(2)-Mn(1)-N(1)	88.15(9)	N(3)-Mn(1)-N(1)	72.95(9)
O(1)-Mn(1)-N(1)	147.15(9)	C(7)-O(1)-Mn(1)	111.71(18)
C(14)-O(2)-Mn(1)#1	128.0(2)	C(14)-O(2)-Mn(1)	125.4(2)
Mn(1)#1-O(2)-Mn(1)	102.99(9)	C(15)-O(3)-Mn(1)	121.2(2)
C(1)-N(1)-C(5)	119.0(3)	C(1)-N(1)-Mn(1)	127.6(2)
N(4)-N(3)-Mn(1)	119.31(19)	C(7)-N(4)-N(3)	109.4(2)
C(3)-C(2)-C(1)	118.5(4)	C(9)-C(8)-C(7)	122.0(3)
Symmetry transformations used to generate equivalent atoms: #1 -x,-y+1,-z			

Table S3 Hydrogen-bonding interactions for **1**.

		**1**		
D-H...A	d(D-H)	d(H...A)	d(D...A)	∠(DHA)
N(2)-H(2C)...O(3)#2	0.86	2.11	2.857(3)	145.5
Symmetry transformations used to generate equivalent atoms: #1 -x,-y+1,-z; #2 -x,-y+2,-z				

Table S4 Selected bond lengths (Å) and bond angles (°) for **2**.

	**2**		
Mn(1)-O(1)	2.129(5)	Mn(1)-O(4)#1	2.150(5)
Mn(1)-O(3)	2.209(4)	Mn(1)-N(1)	2.221(5)
Mn(1)-O(5)	2.275(5)	Mn(1)-N(4)	2.336(6)
O(2)-Mn(1)#1	1.941(2)	O(5)-C(8)	1.248(7)
N(1)-C(1)	1.290(8)	N(1)-N(2)	1.388(8)
N(2)-C(8)	1.347(9)	N(2)-H(2)	0.8600
C(16)-C(17)	1.376(10)	O(2)-C(15)	1.242(9)
C(21)-C(20)	1.381(9)	C(12)-C(13)	1.361(13)
C(9)-C(10)	1.369(10)	O(6)-H(21)	0.8415
O(1)-Mn(1)-O(4)#1	153.77(19)	O(1)-Mn(1)-O(3)	80.40(18)
O(4)#1-Mn(1)-O(3)	93.04(17)	O(1)-Mn(1)-N(1)	100.8(2)
O(4)#1-Mn(1)-N(1)	96.55(19)	O(3)-Mn(1)-N(1)	152.7(2)
O(1)-Mn(1)-O(5)	84.24(19)	O(4)#1-Mn(1)-O(5)	83.24(19)
O(3)-Mn(1)-O(5)	136.37(17)	N(1)-Mn(1)-O(5)	70.35(19)
O(1)-Mn(1)-N(4)	102.3(2)	O(4)#1-Mn(1)-N(4)	102.1(2)
O(3)-Mn(1)-N(4)	83.32(18)	N(1)-Mn(1)-N(4)	69.7(2)
O(5)-Mn(1)-N(4)	140.05(17)	C(22)-O(3)-Mn(1)	117.9(4)
C(15)-O(1)-Mn(1)	131.4(5)	C(22)-O(4)-Mn(1)#1	106.5(5)
C(8)-O(5)-Mn(1)	115.4(5)	C(1)-N(1)-N(2)	118.0(5)
C(1)-N(1)-Mn(1)	124.5(5)	N(2)-N(1)-Mn(1)	117.5(4)
C(8)-N(2)-N(1)	113.0(5)	C(8)-N(2)-H(2)	123.5
N(1)-N(2)-H(2)	123.5	C(17)-C(16)-C(21)	118.0(6)
C(17)-C(16)-C(15)	120.2(7)	C(1)-N(3)-H(33)	110.1
C(6)-N(4)-Mn(1)	124.3(4)	C(2)-N(4)-Mn(1)	116.1(5)
N(4)-C(2)-C(3)	121.7(7)	C(3)-C(2)-C(1)	121.5(6)
C(2)-C(3)-H(3)	121.4	H(22)-O(6)-H(21)	81.4

Symmetry transformations used to generate equivalent atoms: #1 –x+1,-y+1,-z

Table S5 Hydrogen-bonding interactions for **2**.

		**2**		
D-H...A	d(D-H)	d(H...A)	d(D...A)	∠(DHA)
N(2)-H(2)...O(2)#2	0.86	2.02	2.869(7)	169.2
N(3)-H(33)...O(2)#2	0.85	2.09	2.938(9)	174.2
N(3)-H(33)...O(1)#2	0.85	2.63	3.145(8)	120.6
O(6)-H(21)...O(6)#3	0.84	2.48	2.931(13)	114.3
O(6)-H(22)...O(1)	0.80	2.27	2.986(8)	148.7

Symmetry transformations used to generate equivalent atoms: #1 -x+1,-y+1,-z; #2 -x,-y+2,-z; #3 -x,-y+1,-z
